# Best Nursing Practice: Safe and Inclusive Healthcare Environments for Transgender People: A Systematic Review

**DOI:** 10.3390/nursrep14010022

**Published:** 2024-01-25

**Authors:** Jesús Manuel García-Acosta, Francisco Javier Castro-Molina, Alfredo David Fernández-Martínez, Airam Delgado-Reyes, María Andreína Castellano-Fuenmayor

**Affiliations:** 1 The Canary Islands Health Service, Tenerife, 38071 Canary Islands, Spain; extjgarciaa@ull.edu.es (J.M.G.-A.); alu0101139029@ull.edu.es (A.D.-R.); alu0101098906@ull.edu.es (M.A.C.-F.); 2 Nuestra Señora de la Candelaria School of Nursing, University of La Laguna, 38010 Canary Islands, Spain; 3 Department of Education, Vocational Training, Physical Activity and Sport, Regional Government of the Canary Islands, Tenerife, 38010 Canary Islands, Spain; afermar1@gobiernodecanarias.org

**Keywords:** health services for transgender persons, comprehensive healthcare, transgender persons, nurse’s role, advanced practice nursing

## Abstract

(1) Background: The aim of this study was to review the scope of the existing scientific literature on creating safe and inclusive healthcare environments for transgender people and provide an overview of the resources and nursing skills required to do so. (2) Methods: With the research question in mind, an exploratory search of six databases was conducted to identify all relevant primary studies. After screening and selection of articles based on the inclusion and exclusion criteria, a total of 41 articles were included and reviewed. (3) Results: The results were classified under four headings: the training of health professionals, the creation of safe spaces, the nurse as facilitator, and best care practice. Most of the evidence indicates that it is essential for nurses and other healthcare staff to be trained in specific skills to provide comprehensive, high-quality care to transgender people; however, there is a lack of material and human resources to do so. (4) Conclusions: The trans-inclusive care competent nurse should use neutral language that respects the person’s preferred name and pronouns in a safe healthcare environment that offers and ensures warmth, respect, and inclusivity in the care provided. This study was registered with the Open Science Framework (OSF) on 9 January 2024 (osf.io/rpj6a).

## 1. Introduction

Transsexuality became a mental disorder in 1980 when it was first listed in the third edition of the Diagnostic and Statistical Manual of Mental Disorders (DSM-III) [1,2] and was classified under the heading of ‘Disorders Usually First Diagnosed in Infancy, Childhood, or Adolescence’, differentiating it from ‘Gender Identity Disorder of Adolescence or Adulthood, non-Transsexual type’, which would gain more prominence in the scientific literature under the broader term transgenderism [2,3]. In 1978, the World Health Organisation (WHO) included transsexuality in its International Classification of Diseases (ICD-9) in the gender identity disorders category under the term *transsexualism*, which replaced the term *transvestism* included in the previous edition (ICD-8) [4,5].

These manuals are continually being amended, and since 2018, the WHO no longer considers transgenderism a disease but a ‘gender discordance’, among other sexual health conditions [3,6,7]. However, in the latest revision of the DSM-5 (DSM-5-TR), published in 2022, the diagnostic label of gender dysphoria is still considered a psychiatric condition [3,8,9].

This pathological status has persisted over the years, making it difficult to disassociate the concept of transgenderism from that of disease, with serious implications for the trans population, who are regarded as mentally ill patients [7].

Several studies have sought to calculate the prevalence of transgenderism in society without reaching a firm conclusion. The latest version of the Standards of Care for Transgender and Gender Diverse People (2022) estimates the proportion of transgender and gender-diverse people to be between 0.02% and 0.03% [9]. Other authors, however, estimate the prevalence of transgender people in society to be between 0.3% and 0.5% of the total population [10,11,12], meaning that there are currently around 25 million trans people worldwide [11,13].

When analysing the healthcare received by trans people, and despite the fact that the right to health has been enshrined as a human right since 1948, we identified a number of healthcare barriers [14,15]. These include access to or availability of health services due to administrative issues, lack of knowledge of terminology or processes by professionals, not respecting the name and pronouns by which a patient wishes to be called, and being seen in non-inclusive environments that favour marginalisation [16,17,18,19].

Therefore, improving access to healthcare for trans and gender-diverse people requires professional training and a change in institutional health policies. This will help to avoid systematised social determinants [20] such as the discrimination, oppression, marginalisation, and violence that these individuals experience in their communities [20,21], thus contributing to improved safety, self-esteem, mental health, and overall well-being [11]. Consequently, the nurse plays a key role when first contacting patients and will, therefore, be a key player in the care process for trans people [21].

Transgender Europe (TGEU) is a European organisation established in 2005 and is the current human rights reference for the trans community in Europe and Central Asia. It continuously analyses the human rights and health aspects of transgender people.

In this sense, in 2022, they published the first edition of the Trans Health Map, which shows the general availability and accessibility of healthcare according to the type of care and available coverage; psychiatric diagnosis requirement; waiting time for the first appointment; excluded groups with longer waiting times for care; age for blockers; and age for hormone treatment. As the paper notes, transgender people continue to face barriers to access and care [22].

When looking at the rights of trans people in the European Union, 41 of the 54 countries reviewed have administrative or legal measures that recognise trans identities. Of those 41 countries, 28 require a psychiatric diagnosis and 11 require sterilisation. There are only five countries that prohibit conversion therapies on the grounds of gender identity [23].

In light of the above, the aim of this review was to identify the recommendations for advanced nursing practice contained in the available scientific evidence on the care of trans people to create inclusive healthcare environments where these individuals will feel safe, respected, and supported, while seeking to provide comprehensive and holistic care. In this document, the term *trans* is used as an inclusive term for diversity related to gender identity.

## 2. Materials and Methods

### 2.1. Design

A review of the scientific literature was conducted based on the criteria established by the 2020 PRISMA (Preferred Reporting Items for Systematic Reviews and Meta-Analyses) statement [24].

The inclusion criteria for the articles were being published in the past five years; being open access; being related to nursing; and being written in Spanish and/or English. These languages are two of the most widely used languages in scientific and medical production worldwide. By limiting the review to these languages, a wide range of scientific and medical literature can be accessed, thus increasing the possibility of covering a large number of relevant studies. Exclusion criteria were also established for studies that dealt with barriers to healthcare; discriminatory attitudes; mental health disorders; drug use; sexually transmitted infections; ethics; religion; and studies that were not related to the study topic.

In line with the main study objective, the following question was formulated using the PICO format: ‘What are the nursing practices required to create inclusive healthcare environments in accordance with nursing skills in the care of trans people?’ Thus, the population corresponds to trans people, the intervention concerns nursing practices for the creation of inclusive environments, the comparison was made with non-inclusive environments, and the outcomes are nursing skills in the care of trans people.

Committed to making the systematic review protocol rigorous and transparent, pre-registration was sent to the International Prospective Register of Systematic Reviews (PROSPERO). However, given the inexperience with the platform, PROSPERO ultimately did not accept the protocol because it already presented results at the time of registration. Finally, this study was registered with the Open Science Framework (OSF) on 9 January 2024 (osf.io/rpj6a).

### 2.2. Search Strategy

The search took place between January and July 2023 in the following databases: Web of Science (WoS), Virtual Health Library (VHL), Cumulative Index to Nursing and Allied Health Literature (CINAHL), PubMed, Scientific Electronic Library Online (SciELO), and CUIDEN. Studies were identified using the following descriptors from the DeCS/MeSH thesauri: ‘Políticas Inclusivas de Género/Gender-Inclusive Policies’ OR ‘Servicios de Salud para las Personas Transgénero/Health Services for Transgender Persons’ OR ‘Atención Integral de Salud/Comprehensive Health Care’ OR ‘Estrategias de eSalud/eHealth Strategies’ AND ‘Personas Transgénero/Transgender Persons’ AND ‘Rol de la Enfermera/Nurse’s Role’. Table 1 shows the initial results of the search equations.

### 2.3. Data Extraction and Synthesis

The review process was carried out in two phases. In the first phase, a literature search was conducted in the various databases. Articles were screened by first reading the title and then reading the abstract and, if the article was considered eligible for inclusion in the literature review, its content was analysed to identify any inclusion or exclusion criteria. In the second phase, other literature sources were reviewed and selected through a reverse reference search to broaden and deepen the scope of the data retrieved, thus providing greater coverage of the research objective. The inclusion criterion ‘year of publication’ was not considered for the sources retrieved through a reverse reference search.

Both the search and the analysis of the data sources were carried out by two researchers. In case of disagreement on eligibility, a third researcher was appointed to make the final decision. This third researcher acted as an impartial and objective arbiter to resolve discrepancies and make a final decision on the inclusion or exclusion of an article in the review. Thus, he was responsible for independently reviewing the disputed article; considering the arguments of the initial reviewers; making an objective decision; and facilitating consensus, if possible.

### 2.4. Quality Appraisal

The level of evidence (LE) and grade of recommendation (GR) of each selected article were assessed based on the Scottish Intercollegiate Guidelines Network (SIGN) guidelines [25], and their quality (Q) was appraised using the Critical Appraisal Skills Programme Español (CASPe) instruments [26].

The criteria for LE and GR analysis using the SIGN guidelines have been improved twice; the first time in 2000 to conform to the LEs proposed by the Agency for Healthcare Research and Quality (AHRQ) and the second in 2009 to implement the GRADE approach [27].

## 3. Results

**Figure 1:**
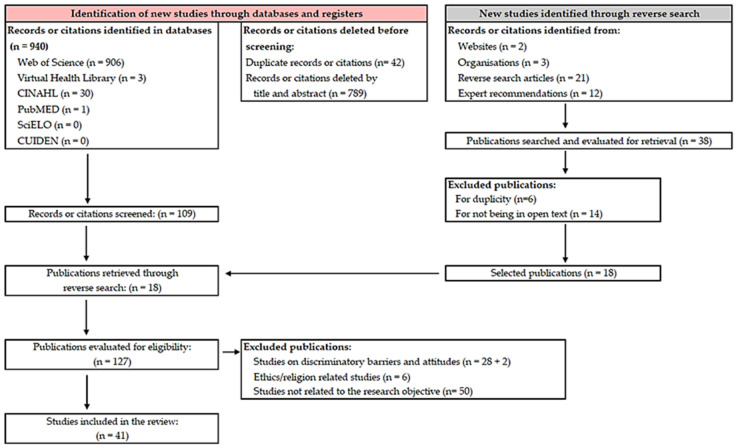
Flowchart of the review. Adapted from the PRISMA 2020 statement.

Our analysis of the available scientific evidence allowed us to retrieve a total of 940 articles. The use of Refworks^®^ (ProQuest RefWorks 2.0, 2010) reference management software and the Covidence^®^ (Covidence systematic review software, Veritas Health Innovation, Melbourne, Australia) screening and data extraction tool for conducting systematic reviews made it possible to eliminate 42 duplicates. A total of 18 additional documents were retrieved through the reverse literature search. Finally, once the eligibility and exclusion criteria were established, the total number of articles to be reviewed was 41, as shown in the flowchart (Figure 1).

The included studies were conducted in the following countries: the United States (n = 23), Brazil (n = 8), Canada (n = 4), Costa Rica (n = 1), Georgia (n = 1), Mexico (n = 1), Sweden (n = 1), the United Kingdom (n = 1), and Uruguay (n = 1). The quality assessment results are shown in Table 2. The included studies are summarised in Appendix A.

### 3.1. Training of Health Professionals

To be able to always ensure and guarantee compliance with human rights and equity, all nurses, other healthcare professionals, and administrative staff working at healthcare facilities must be trained and educated in gender diversity [9,15,20,28]. Accordingly, nurses must have an academic background with a master’s degree or equivalent qualification in sexology or psychology in which they should have acquired non-pathologising communication skills while receiving continuous training in transgender issues and working on transphobia-related aspects [29].

It is essential for nurses to properly understand and manage the needs of and specific care required by trans people in order to provide a comprehensive, standardised, inclusive approach that is tailored to each individual [30]. That said, multiple studies report that training for health professions should focus on learning the associated terminology, the proper use of nouns and pronouns, raising awareness, and identifying negative attitudes, such as stigma and prejudice, thus fostering the development of a good therapeutic relationship [11,30,31,32,33,34,35,36,37,38,39]. Failure to respect the name a person identifies with may lead to feelings of shame and humiliation that may result in the person avoiding seeking healthcare in the future [15,40]. In many instances, the lack of matching legal documents facilitates transphobic abuse and discrimination [41].

In line with the latter, Russell et al. reported that the use of the name that they identify with decreases suicidal ideation by up to 29% and suicidal behaviour by up to 56% [38], as family construction and functioning can be complex and provides little scope for action [42].

Several studies demonstrate that the use of culturally sensitive, informed language not only reduces the number of micro-aggressions but also strengthens the relationship between health workers and the population they serve [43,44]. Thus, studies such as the one conducted by Koch et al. illustrate the utility of training programmes for nurses in social and communication skills through active methodologies, such as role play [8].

### 3.2. Creating Safe Spaces

A safe space is one in which trans people can express themselves naturally [11] and which is perceived as friendly, inclusive, and respectful [45]. Results show that the use of same-sex couples and rainbow flags on posters, magazines, brochures, stickers, and/or flyers in waiting areas can facilities makes environments more welcoming by showing staff awareness, inclusion, and respect for this community [11,45,46,47,48].

This requires effort and commitment on the part of health professionals and institutions to understand the desires and expectations of these users and to make the necessary resources available to achieve these goals and meet their healthcare needs [8,49]

Guidelines and training programmes have been developed to make healthcare settings more welcoming and define not only which factors make them welcome but also how healthcare professionals can effectively implement such factors to achieve the best health outcomes for trans people accessing these settings [46].

### 3.3. The Nurse as Facilitator

In most settings, nurses are the first health professionals that patients come into contact with, which is both a great opportunity and a challenge when providing gender-affirming care. It is a significant encounter that sets the tone for these individuals’ future experiences within healthcare settings [11,28,50].

A proper nursing approach requires seeing patients as biopsychosocial and cultural beings and it must take the characteristics of each individual into consideration along with their specific needs, as well as their health expectations within their social and cultural context [14]. Emphasis should be placed on getting to know the patient as a person and treating them with empathy rather than as a medical case or condition [38].

Nurses can also implement community practices to facilitate transgender awareness and normalisation in society (e.g., workshops on sexual and gender diversity education or patient school lectures), enabling trans people to express their gender identity in a way that is safe for their health [9,51] while promoting their engagement as key drivers of change [46].

In addition, nurses can facilitate and foster understanding and acceptance of trans individuals within their families [46] (e.g., by offering psychological support and counselling resources, such as associations), which is of utmost importance. For instance, a study by Rafferty et al. found that rates of suicidal ideation among trans adolescents ranged from 4% in those with strong family support to 60% in those without family support [42].

Nurses can also work as case managers by advocating for the multidisciplinary approach proposed by the World Professional Association for Transgender Health (WPATH) in its manual entitled Standards of Care for Transgender and Gender-Diverse People [9]. As such, trans people’s healthcare is the responsibility of a broad team of primary care physicians, nurses, surgeons, psychologists, and endocrinologists, among other healthcare providers. Moreover, to ensure successful care and medical attention, all these professionals must keep in close contact to meet their patients’ desires and expectations [9,35,52].

Another important aspect for which nurses are responsible is the promotion of individual autonomy and self-care, which is a fundamental aspect of healthcare. Nurses must play their role as facilitators by supporting and allowing patients to achieve their own goals [52,53], making them responsible for their own health, and encouraging their active involvement in their health-related decisions [9,41], as many of them may undergo procedures that may or have irreversible repercussions [35].

### 3.4. Best Care Practices

Trans patients should be given the opportunity to describe their own identity rather than have others assume it [54], with professionals always using neutral language [36,48] and avoiding any presumption of a patient being cisgender and heterosexual [45]. Also, the person’s preferred name and pronouns should be respected, thus ensuring that the care provided is welcoming, respectful, and inclusive [10,30,37,41,49,55,56]. Similarly, every effort should be made to avoid focusing on gender-related issues that are unrelated to the actual reason for consultation [38].

Health professionals should also focus on promoting health and well-being, not just reducing potential dysphoria [10,49,50]. The reason for their clinical assessment and how relevant this assessment is for providing these patients with high-quality care must be explained to them [41]. In all cases, health professionals are responsible for ensuring the confidentiality of all data obtained, of which users must also be made aware [38].

When taking their history, open-ended and generic questions using inclusive and neutral language should be asked [29,36,37,57], and users should have the option of not answering certain questions or postponing their assessment until the therapeutic relationship becomes stronger [41]. A comprehensive, professional, non-judgemental interview regarding sexual and gender identity should be conducted with all patients as part of routine history taking and physical examination, including screening for depression and other mental health issues [38]. For instance, if the health professional were to ask about their sexual health, they could use questions such as ‘Are you in a relationship with someone?’ ‘Do you have questions about your sexual health?’ ‘What do you think about your risk of contracting sexually transmitted infections or about pregnancy?’ [45,55]. Health professionals should also ask about their desire to receive hormone therapy or, if they are already on it, have them describe their administration technique. This may be a good opportunity to assess the safety of drug administration, bearing in mind that it is inappropriate to give feedback on the person’s physical appearance and body changes [55].

They should be asked what terms they are most comfortable using to talk about their genitalia (‘pelvis’ can be used as a neutral term) and asked whether they would like to have upper or lower surgery, rather than assuming they do [30,55]. An anatomical model can also be used [41].

Finally, it is important to note that special care should be taken during physical examinations. If the health professional needs to perform a physical examination, they should do so only when strictly necessary, continuously assessing the person’s comfort and willingness to undergo the examination [55,58,59] and ensuring that the person remains in control at all times [60]. In line with this, multiple studies report on the benefits of having a supportive trusted person present during the examination process [55,61].

## 4. Discussion

Today, trans people continue to face pathologisation, discrimination, negative attitudes, prejudice, and social stigma that affect them beyond their social sphere. This social stigma, in particular, continues to surround trans people, generating vulnerability and health risks. Fear of discrimination, lack of appropriate healthcare, and poor quality of life lead them to resort to other options outside health services, which are often completely unsuitable for them. Suicide and suicidal ideation figures should be approached with caution, taking intersectionality into account. Thus, in addition to varying with age and family support [42] or the use of a meaningful name [38], there are other variables related to cultural identity (race/ethnicity and religion) and the existence of psychiatric comorbidities, which must be taken into account [62]. In this sense, a systematic review by Wolford-Clevenger et al. found that white transgender people with lower levels of acceptance of their religious identity and a history of mental health illness were more likely to attempt suicide [62].

Many studies address the way in which such barriers to healthcare occur, which translates into specific health risks for trans people and can, as a result, worsen their quality of life by excluding them from participation in disease prevention and health promotion programmes like any other citizen [15,16,18,19,28,39,57,59]. Therefore, nurses need to support the creation of safe and inclusive spaces for trans people in order to improve the quality of the healthcare that these individuals receive, while promoting better health and well-being in this population.

The creation of safe and inclusive spaces for trans people by nurses offers a variety of benefits for this community. Such spaces enable trans people to feel comfortable when attending scheduled appointments in healthcare facilities where guidance and resources are available, create a trusting therapeutic relationship between them and nurses and other healthcare professionals, and improve the quality of health and well-being in the trans community.

While there are increasing resources available on how to create welcoming and safe healthcare environments [46], it is important to note the importance of taking into account another essential component of nursing education: namely LGBTQ+ cultural competence. Future educational guidelines should integrate the social and cultural contexts of sexual and gender minorities [63].

Most of the available evidence reports the need to equip nurses and other healthcare workers with specific skills to provide high-quality holistic care for trans people based on trust, respect, and inclusivity [11,14,28,29,32,33,39,43,44,55,61]. However, only a few studies present learning tools and recommendations to do so. It would, therefore, be interesting to explore this field in more depth to make nursing interventions more robust [9,28,29,38,46,49,55].

Furthermore, nurses must be adequately trained in understanding and respecting the trans community. As such, training should begin at the undergraduate level, and postgraduate training programmes should be implemented that include both the identification of risk factors in the trans community and the management of sensitive situations regarding healthcare practices and mental health support. These two aspects are particularly important, as the lack of information and training is one of the main obstacles to healthcare and leads to pathologised healthcare [20,30,40,45,61], with many users reporting discriminatory attitudes, abuse, or outright harm when receiving healthcare [45].

Another relevant consideration is that the database search only produced one study reporting the results of a training exercise on nurse skill acquisition, including social and communicative skills, to improve their therapeutic relationship with trans people. It would, therefore, be desirable to conduct further studies that address the implementation of training programmes that equip nurses with the necessary tools to provide appropriate healthcare for trans people in greater depth [8].

Finally, it should be pointed out that despite the fact that specific training guidelines and programmes have been developed for healthcare professionals on this topic [9,29,41], our database search only produced one study that highlights the need to develop a solid body of evidence on the following aspects: the long-term effects of hormones on trans people, as well as the effects of puberty suppression to which they may be subjected; the different types of plastic surgery procedures that trans people may have undergone and their effects on their bodies; the social determinants that favour specific types of discrimination against trans people; pregnancy and breastfeeding/chestfeeding among trans people; and the relevance of inclusive and exclusive care in healthcare facilities, among others [46].

This systematic review has some limitations that should be considered when interpreting the results. First, the selection of studies may have been biased, as only articles published in English and Spanish were considered. There is a possibility that some relevant studies may not have been identified, thus introducing a linguistic or geographical bias in the review results. Next, the variable methodological quality of the studies included in the review may influence the reliability of the results. Finally, differences in study design, population, interventions, or outcomes may make data synthesis and direct comparison difficult, limiting the ability to draw clear and definitive conclusions.

## 5. Conclusions

The literature review reveals that there are multiple variables that influence the health and well-being of trans people beyond factors such as age, family support, or the use of a name that reflects their identity. Cultural elements such as race, ethnicity, and religion, as well as the presence of additional psychiatric conditions, are crucial factors that should be considered in future research.

In this context, the role of nurses is vital. A competent nurse must provide an approach based on biopsychosocial and cultural care that considers the particular needs of each person.

The nurse, as a facilitator within the interdisciplinary team, should be a trained health professional who understands the gender diversity, specific needs, and challenges of trans people. The nurse should be aware of the impact of discrimination and lack of access to healthcare in the national healthcare system, as well as the available treatment options offered within the service portfolio. In addition, they must acquire the following necessary skills: effective communication; using neutral and respectful language that offers and ensures warmth, respect, and inclusivity in the care provided; decision making in individualised care; and they must be motivated for lifelong learning in this field.

Nurse education and training must be a priority in a changing society. From risk identification to the management of sensitive situations and mental health support, institutions have a key role to play in the training of health professionals. It is essential to provide educational programmes that address these needs, as lack of information and training is one of the main barriers to adequate healthcare.

Nevertheless, despite current efforts, there is a clear need for more research in this field. Aspects such as the long-term effects of hormones, plastic surgery, discrimination, pregnancy, and breastfeeding in transgender people need to be explored in order to develop a solid body of evidence.

## Figures and Tables

**Table 1:** Search results.

Database	Filters Used	Results
Web of Science (WoS)	Last 5 years Full text Spanish and English Area: Nursing	906
Virtual Health Library (VHL)	None	3
Cumulative Index to Nursing and Allied Health Literature (CINAHL)	Last 5 years Full text Spanish and English	30
PubMed	Last 5 years Spanish and English	1
Scientific Electronic Library Online (SciELO)	Last 5 years Area: Nursing	0
CUIDEN	None	0

**Table 2:** Quality appraisal.

Author	Citation	Year	LE	GR	Q
Aarne-Grossman, V.G.	[34]	2016	2++	B	6/10
Abreu, P. et al.	[56]	2022	4	D	10/10
Bernstein, S.M. et al.	[47]	2018	4	D	5/10
Buchholz, L.	[12]	2015	3	D	9/10
Carlström, R. et al.	[57]	2021	4	D	10/10
Castilla-Peón, M.F.	[61]	2019	3	D	8/10
Collins, C.A.	[40]	2021	3	D	9/10
Conron, K.J. et al.	[14]	2012	2+	C	11/11
Costa, A.B. et al.	[16]	2018	4	D	10/10
Danilo-Fagundes, R. et al.	[7]	2019	3	D	9/10
Florêncio, L.L.F. et al.	[20]	2021	4	D	9/10
Guss, C.E. et al.	[58]	2019	4	D	8/10
Hana, T. et al.	[21]	2021	2+	C	6/10
Hancock, A.B. et al.	[17]	2021	2+	C	9/10
Hunt, R. et al.	[45]	2019	2++	B	10/10
Imborek, K. et al.	[37]	2017	3	D	5/10
Karasic, D.H. et al.	[35]	2018	3	D	5/10
Klein, D.A. et al.	[53]	2018	3	D	6/10
Koch, A. et al.	[8]	2021	2++	B	11/11
Levitt, N.	[33]	2015	2+	C	6/10
Michels, S. et al.	[44]	2020	2+	C	8/10
Nascimento, F.K. et al.	[3]	2020	4	D	10/10
Núñez, V. et al.	[59]	2022	1++	A	10/10
Potter, J. et al.	[60]	2015	2+	C	7/10
Puckett, J.A. et al.	[18]	2018	2+	C	11/11
Rafferty, J. et al.	[42]	2018	2++	D	6/10
Redfern, J. et al.	[38]	2014	2++	B	10/10
Rider, G.N. et al.	[31]	2019	4	D	10/10
Rigolon, M. et al.	[39]	2020	4	D	10/10
Santos, J.S.D. et al.	[15]	2019	2++	B	7/10
Safer, J.D. et al.	[19]	2016	2+	C	7/10
Silva, N.L. et al.	[43]	2021	2++	B	9/10
Soto-Rodríguez, M.A.	[1]	2014	3	D	6/10
Sundus, A. et al.	[30]	2021	2++	B	10/10
Veale, A. et al.	[46]	2022	4	D	10/10
Weiselberg, E.C. et al.	[48]	2019	2+	C	7/10
Winter, S. et al.	[11]	2016	2++	B	8/10
Wolfe-Roubatis, E. et al.	[32]	2015	3	D	9/10
Ziegler, E.	[10]	2021	4	D	8/10
Ziegler, E. et al.	[50]	2021	2−	D	8/10
Zimmerman, A.R. et al.	[51]	2020	2++	B	7/10

1++ Meta-analyses (MA), high quality, systematic reviews (SR) of clinical trials or high quality clinical trials with very low risk of bias. 2++ High-quality SRs of cohort or case-control studies. Cohort or case-control studies with very low risk of bias and with a high probability of establishing a causal relationship. 2+ Well-conducted cohort or case-control studies with low risk of bias and with a moderate probability of establishing a causal relationship. 2− Cohort or case-control studies with high risk of bias and significant risk that the relationship is not causal. 3 Non-analytical studies, such as case reports and case series. 4 Expert opinion. This legend is taken from the Scottish Intercollegiate Guidelines Network (SIGN) guidelines [25].

## Data Availability

The data presented and pre-registration details of this study are available upon request from the corresponding author.

## Appendix A

**Author**	**Year**	**Country**	**Study Design**	**Aim**	**Results**
Aarne-Grossman, V.G. [34]	2016	USA	Conceptual review	To understand how to address and manage the health needs of transgender people.	Results were grouped under the headings organisational sensitivity, health personnel, suggestions for care, and radiological tests.
Abreu, P. et al. [56]	2022	Brazil	Qualitative	To analyse comprehensive healthcare for transgender adolescents from the perspective of their guardians.	There are difficulties in accessing services. The lack of preparation of professionals is pointed out, and the importance of adequate reception and emotional support is emphasised.
Bernstein, S.M. et al. [47]	2018	Georgia	Panoramic review	To identify tactics to support trans users and families within consultations.	Results were grouped under the headings introduce yourself and what you prefer to be called, ask open questions, encourage other community members to take a stand, and facilitate inclusive environments.
Buchholz, L. [12]	2015	USA	Case report	To describe the experience of care.	Health professionals need to be continuously trained in transgender research. There is a lack of training in university curricula.
Carlström, R. et al. [57]	2021	Sweden	Qualitative	To describe the experiences of transgender people with healthcare providers.	Transgender people demand respectful treatment and care according to their health needs. Professionals question their identity and lack adequate training.
Castilla-Peón, M.F. [61]	2019	Mexico	Panoramic review	To provide an overview of the important aspects to consider in the medical management of transgender children.	Outcomes were grouped under the headings epidemiology; healthcare for transgender children and adolescents; gender transition and mental health; endocrinological interventions; surgical interventions; and reproductive aspects.
Collins, C.A. [40]	2021	USA	Cross-sectional descriptive	To determine the attitudes, beliefs, knowledge, and perceived competence of paediatric nurses in caring for transgender youth.	A total of 85% of the nurses had not received any formal training, and 65% had taken a continuing education course. No correlation was found between age and years of experience versus attitudes and competence, but there was a significant correlation with training.
Conron, K.J. et al. [14]	2012	USA	Observational analytical	To provide estimates of various indicators of health and socio-economic status by transgender status in a representative sample of households.	The worst health rates were found in transgender people with HIV, high rates of unemployment, and poverty. Despite greater inequalities, they have an average or higher level of education.
Costa, A.B. et al. [16]	2018	Brazil	Observational analytical	To identify the specific care needs of transgender and gender-diverse people.	More than 50% of respondents feel uncomfortable discussing their health needs with health professionals. Thirty percent of them had to tell professionals about their specific health needs.
Danilo-Fagundes, R. et al. [7]	2019	Brazil	Systematic review	To describe and analyse the national and international scientific publications on nursing care for the transgender and/or gender-diverse population.	The studies were categorised into gaps in care for transgender people, transgender population health, general and specific demands, and health policies for transgender people.
Florêncio, L.L.F. et al. [20]	2021	Brazil	Qualitative	To discuss the therapeutic pathway of trans people seeking healthcare from the user’s point of view.	Comprehensive care for transgender people was divided into four categories: (a) low demand for transgender people in health services, (b) use of their social names in health services, (c) prejudiced and discriminatory attitudes, and (d) health system and professionals unable to address health problems.Pathologisation and discrimination are the biggest challenges. Healthcare is avoided to avoid embarrassing situations.
Guss, C.E. et al. [58]	2019	USA	Qualitative	To learn about the primary care experiences of transgender adolescents and their recommendations for primary care practices and physicians.	In general, experiences are positive, although they are concerned about privacy of identity and not respecting the name and pronoun meaning. Professionals must be prepared to deal respectfully and appropriately with any type of person.
Hancock, A.B. et al. [17]	2021	USA	Qualitative	To collect and analyse the perspectives of people from racial and gender minority groups and establish barriers related to voice.	A total of 40% reported feeling accepted by their close environment and 29% by society. A lack of training related to transition was reported.
Hunt, R. et al. [45]	2019	United Kingdom	Systematic review	To evaluate all relevant materials related to the education of health professionals on LGBTI care with a focus on improving training.	Training projects ready to be implemented or useful materials for the training of professionals on LGTBIQA+ issues are collected.
Imborek, K. et al. [37]	2017	USA	Panoramic review	To understand the impact and extent of the use of the preferred name and pronoun and gender identity in the electronic health record.	Results were grouped under the headings preferred name implementation, challenges related to name implementation, technical challenges in the electronic health record, and interpretation of laboratory tests.
Karasic, D.H. et al. [35]	2018	USA	Panoramic review	To describe the key competencies between health professionals and users of the health system.	All professionals must meet a set of competencies, grouped into four domains: caregiver–recipient relationship, content knowledge, interdisciplinary practice and accountability, and professional ethics. Health professionals must be familiar with transgender health settings and teamwork is essential.
Klein, D.A. et al. [53]	2018	USA	Conceptual review	To identify the recommendations for practice in caring for transgender and gender-diverse people.	Outcomes were grouped under the headings establishing an optimal clinical environment, assessment (medical history and physical examination), mental health, health maintenance, hormone therapy and surgery, and other treatments.
Koch, A. et al. [8]	2021	USA	Experimental	To increase students’ knowledge and comfort with caring for a transgender person.	The nursing students reported that they felt comfortable with the program and with learning how to communicate with trans patients and their families.
Levitt, N. [33]	2015	USA	Panoramic review	To learn about ways in which nurses and other health professionals can help create a transgender-sensitive environment.	Nurses play a significant role in health promotion and cancer prevention. Nursing education is an essential foundation, particularly in the field of oncology.
Nascimento, F.K. et al. [3]	2020	Brazil	Qualitative	To describe attributes associated with the quality of life of Brazilian transgender children and adolescents based on their own perceptions.	Results were grouped into five domains. The nuclear family was identified as the main social support for transgender people. However, experiences of prejudice and discrimination were negative attributes associated with quality of life.
Núñez, V. et al. [59]	2022	Uruguay	Systematic review	To provide tools for providing adequate support and follow-up in the healthcare of trans children and adolescents at the first level of care.	Guidelines for the first level of care for trans children and adolescents were developed, providing tools for the clinical history, taking the interview, the physical examination, and a multi- and interdisciplinary approach into consideration.
Potter, J. et al. [60]	2015	USA	Conceptual review	To identify the resources and recommendations for improving the sexual health of transgender men.	Examples of care and key concepts to consider with trans men. Provides guidelines for pelvic examination and the use of language in consultations.
Puckett, J.A. et al. [18]	2018	USA	Observational analytical	To better understand the barriers to gender-affirming care for transgender people.	A statistical association was found between gender and the level of participation, with more transgender women participating. More than 60% of participants were on hormone treatment, with transgender women having a higher rate. About 82% received blockers at puberty. They reported that there were problems at the centres with continuing hormone treatment and the possibility of surgery.
Rafferty, J. et al. [42]	2018	USA	Systematic review	To review the most relevant concepts and challenges for professionals in the field of paediatrics.	Results were grouped under the headings definitions, epidemiology, mental health implications, gender affirmative care, developmental considerations, health environments, pubertal suppression, gender affirmation, health disparities, family acceptance, and safe schools and communities.
Redfern, J. et al. [38]	2014	USA	Systematic review	To examine communication and procedural barriers to care for transgender people.	Practical resources for health professionals to improve interactions and communication with transgender people.
Rider, G.N. et al. [31]	2019	USA	Qualitative	To examine healthcare providers’ experiences and attitudes about working with transgender youth in order to identify specific training needs.	Five themes characterised the responses to the interview questions: (1) training in gender diversity, (2) discomfort with gender issues, (3) reasons for not asking about gender, (4) talking about gender with patients, and (5) need for resources. The themes and sub-themes are developed below, illustrated with representative quotes.
Rigolon, M. et al. [39]	2020	Brazil	Qualitative	To better understand the life histories and pathways of transvestites and transgender people in health services.	Two themes emerged: (1) gender and sexuality in life histories and (2) trajectories in health services. These revealed the challenges in the process of recognising gender identity in front of family and society. The stories illustrate the dilemmas that transsexuals and transvestites face in healthcare, which ultimately lead to the alienation of this population from health services.
Santos, J.S.D. et al. [15]	2019	Brazil	Panoramic review	To reflect on approaches to healthcare for the LGBTI+ population in primary healthcare and nursing care for this population.	In family health teams, nurses should be aware of the main demands of this population. The institutional reorientation of primary healthcare poses new challenges to making the right to healthcare for the LGBTI+ population effective.
Safer, J.D. et al. [19]	2016	USA	Conceptual review	To briefly review the literature characterising barriers to healthcare for transgender people and propose research priorities for understanding the mechanisms of these barriers and interventions to overcome them.	Priorities for research on barriers should include the identification of knowledge gaps across the range of training, possible interventions for those gaps, identification of indirect barriers, such as environment and stigma, and possible solutions to overcome those barriers.
Silva, N.L. et al. [43]	2021	Brazil	Panoramic review	To analyse the concept of the social identity of the transgender person and develop a nursing diagnosis related to the social identity of the transgender person.	Critical attributes, history, and consequences for the social identity of the transgender person were identified, and the analysis of the concept served as the basis for the diagnostic proposal: a provision for the improvement of the social identity of the transgender person.
Soto-Rodríguez, M.A. [1]	2014	Costa Rica	Panoramic review	To analyse the construct of gender identity and its pathologisation throughout history.	Results were grouped under the headings the construction of certain gender identities as psychopathological categories, political resistance and the paradoxical case of certain trans experiences, and the support group as a space in resistance: a place to subjectivise.
Sundus, A. et al. [30]	2021	Canada	Panoramic review	To synthesise the literature and identify gaps in approaches to the provision of ethical and culturally competent transgender care.	There is an apparent dearth of literature on the ethical and culturally sensitive care of transgender people. The review identified that health professionals need to educate themselves about sensitive issues, be more self-aware, put transgender people in charge during care interactions, and adhere to the principles of advocacy, confidentiality, autonomy, respect, and disclosure.
Veale, J. et al. [46]	2022	USA	Qualitative	To highlight issues that need further research and outline key considerations for those conducting research in this field.	The field of trans health research should also have a broader focus beyond medical transition or gender affirmation, including general health and routine medical care; the lives of trans people without, before, and after medical gender affirmation; and sexuality, fertility, and reproductive health needs.
Weiselberg, E.C. et al. [48]	2019	USA	Panoramic review	To identify the general and specific aspects of caring for transgender children and young people.	Results were grouped under the headings terminology, the terminology associated with diagnosis and treatment, stages of gender development, aetiology, diagnostic features of gender dysphoria, disparities in care, mental health, protective factors, and primary care.
Winter, S. et al. [11]	2016	USA	Panoramic review	To examine the social and legal conditions in which many transgender people live and the medical perspectives that shape the provision of healthcare for transgender people in much of the world.	Results were presented in tables and under the headings healthcare in gender affirmation, the size of the trans population, biological correlates in the development of gender dysphoria, rights and health, and trans people—mental disorders?
Wolfe-Roubatis, E. et al. [32]	2015	USA	Qualitative	To understand the needs of transgender men regarding breastfeeding from an experiential point of view.	There is minimal nursing literature on the role of the nurse in transgender healthcare in general, and less is known about the relationship between nurses and transgender male perinatal patients.
Ziegler, E. [10]	2021	Canada	Qualitative	To understand the key nursing activities, training, and support needed to provide primary care to transgender people.	Nurses are important in providing primary care for transgender people. While NPs worked with the full scope of practice, the roles of RNs and RPNs could be optimised. A key challenge was the lack of education; however, mentoring and collaboration contributed to competency development. Ensuring that the workplace provided gender-affirming care was key to a safe and inclusive environment.
Ziegler, E. et al. [50]	2021	Canada	Observational analytical	To develop and implement an educational resource platform to respond to a demand in nursing education related to the concept of cultural humility and its application in health consultations with people who identify as LGBTIQ+.	The Sexual Orientation and Gender Identity Nursing Toolkit was created to promote cultural humility in nursing practice. The toolkit focuses on meetings that use cultural humility to meet the specific needs of the LGBTQI+ and Two-Spirit communities.
Zimmerman, A.R. et al. [51]	2020	USA	Panoramic review	To illustrate how nurses optimise psychosocially, hormonally, and surgically gender-affirming care by conducting comprehensive assessments, coordinating care, and providing health education and training.	Outcomes were grouped under the headings history of gender-affirming care, effective nurse–patient relationships, comprehensive assessments, coaching and health education, and health and well-being.
